# A Wearable Respiratory Monitor for Home-Based Screening and Stratification of Obstructive Sleep Apnea: A Pilot Study in Participants with Intermediate-to-High STOP-Bang Scores

**DOI:** 10.3390/bios16080454

**Published:** 2026-08-20

**Authors:** Burcu Kolukisa Birgec, Beyza Toprak, Alexander Balfour Mullen

**Affiliations:** Strathclyde Institute of Pharmacy and Biomedical Sciences, University of Strathclyde, Glasgow G4 0RE, UK; burcu.kolukisa@strath.ac.uk (B.K.B.); beyza.toprak.2022@uni.strath.ac.uk (B.T.)

**Keywords:** obstructive sleep apnea, biosensor, monitoring, STOP-Bang questionnaire

## Abstract

Access to in-laboratory polysomnography (PSG) is restricted by prolonged waiting lists, and first-night effects can distort typical sleep architecture. This study evaluates the PneumoWave biosensor as a scalable, unobtrusive alternative for longitudinal single-channel home sleep apnea monitoring. Over three nights in an uncontrolled home environment, the biosensor’s respiratory rate agreement was evaluated against smartwatch-derived respiratory rate estimates, whilst apnea/hypopnea detection was compared with concurrent pulse oximetry. PneumoWave and smartwatch devices demonstrated good correlation (*r* = 0.870; *p* < 0.001). The PneumoWave device showed strong measurement agreement and provided a highly predictive screening pathway for patients with intermediate-to-high obstructive sleep apnea (OSA) risk. Longitudinal analysis confirmed consistent multi-night performance without first-night effect biases (ICC = 0.956, *p* < 0.001). Furthermore, its intuitive design yielded zero patient-induced setup errors, highlighting its operational robustness for self-administered use. Combining this continuous chest wall monitor with the STOP-Bang clinical questionnaire has the potential to provide an effective predictive screening pathway, improving community OSA screening and assisting clinical triage. Further validation against full polysomnography is warranted before clinical adoption.

## 1. Introduction

Obstructive sleep apnea (OSA) is a highly prevalent respiratory disorder characterized by recurrent episodes of complete (apnea) or partial (hypopnea) upper airway collapse, leading to intermittent hypoxia and sleep fragmentation [1,2]. OSA affects an estimated one billion adults aged 30 to 69 years globally [3,4]. OSA imposes a profound clinical and socioeconomic burden. Untreated OSA is independently associated with severe cardiovascular and metabolic comorbidities, alongside cognitive dysfunction and mood disorders [2,3,5,6,7]. Furthermore, the indirect consequences of undiagnosed OSA range from increased hospital admissions and emergency interventions to workplace absenteeism and motor vehicle accidents [3,8,9]. Despite this immense public health burden, most cases remain undiagnosed due to low public awareness and structural barriers within healthcare systems that restrict access to diagnostic facilities. The STOP-Bang questionnaire is frequently used as a clinical screening tool for triaging individuals suspected of having OSA who are referred to sleep clinics since it displays adequate sensitivity and diagnostic accuracy for detecting moderate to severe obstructive sleep apnea (questionnaire score ≥ 3) [10,11].

The current clinical gold standard for OSA diagnosis is in-laboratory polysomnography (PSG). While PSG provides comprehensive, multi-channel physiological data encompassing sleep architecture, oxygen desaturation, and respiratory biomechanics [3,12], it is fundamentally limited by high operational costs, long waiting lists, and the requirement for specialized infrastructure and trained technicians. Crucially, single-night PSG often fails to capture the natural night-to-night variability of sleep-disordered breathing. The unfamiliar laboratory environment and the encumbrance of multiple sensors frequently disrupt typical sleep patterns (the so called ‘first-night effect’), which can result in inadequate sleep duration and clinically significant diagnostic misclassification [3,13,14].

To overcome these limitations, recent advancements in wearable biosensor technology have catalyzed a paradigm shift toward continuous, home-based physiological monitoring. Wearables offer a vital opportunity to capture high-fidelity, real-world data longitudinally, thereby minimizing point-in-time diagnostic errors. The recent studies evaluated different types of devices including wearable photonic wristbands for heart, breathing rate, and blood pressure assessment during physical activity linked with an individual’s body mass index (BMI) [15]; 3D printed sensors to monitor breathing [16]; and piezoresistive sensors assessing snoring and head positions during sleep [17].

The PneumoWave biosensor is a UKCA Class I wearable device engineered for the continuous, non-invasive assessment of respiratory dynamics [18,19,20]. Through a dedicated digital dashboard, the system allows researchers access to accelerometer-derived waveform data transparently, which are synthesized from raw chest movements signals utilizing the manufacturer’s proprietary algorithmic framework [21]. The main aim of this study was to evaluate the PneumoWave biosensor in an uncontrolled home environment over multiple nights among individuals with STOP-Bang Scores ≥ 3, indicative of moderate to severe OSA. Specifically, we aimed to determine its viability as a reliable mechanical proxy for detecting and monitoring complex sleep-disordered breathing events, including apneas, hypopneas, and snoring.

## 2. Materials and Methods

The hardware and software system of the PneumoWave biosensor has been previously described [18,19,20]. During analysis, no automated algorithms or scoring systems were utilized. Instead, all apnea and hypopnea events were exclusively manually scored and quantified by the trained researchers.

### 2.1. Participants and Ethical Considerations

The University of Strathclyde’s Ethics Committee approved the study (Reference UEC24/91, February 2025). A priori sample size estimation was conducted to ensure adequate statistical power for detecting physiological differences between the reference standard and the PneumoWave biosensor. Assuming a standard deviation of the measurement differences of 2.0, with an alpha level of 0.05 and 80% power, a minimum of 11 participants was mathematically required. To account for a potential 20% drop-out rate or data loss secondary to sensor detachment during unattended home sleep monitoring, the final target sample size was set at 15 participants.

Before taking part, all participants provided written informed consent. In full compliance with the Declaration of Helsinki, participants were fully briefed on the research aims, procedures, and possible risks, and it was emphasized that their involvement was entirely voluntary, with the right to withdraw at any point without penalty. All participants completed a pre-screening questionnaire to determine eligibility (see Supplementary Material—Recruitment Information), and the data collection, storage, and processing strictly adhered to the General Data Protection Regulation (GDPR; Regulation (EU) 2016/679). To ensure confidentiality, all participant identities were completely anonymized before conducting analysis, and the resulting research data were safely maintained on a secure university cloud server.

### 2.2. Experimental Protocol

Participants completed a STOP-Bang Questionnaire to assess their risk of OSA. They were equipped with monitoring devices to be used for three consecutive nights over a one-week period. These included a PneumoWave biosensor (PW010; PneumoWave, Maxim Park, UK), a wearable smartwatch (Galaxy Watch 4; Samsung, Suwon, Republic of Korea), a portable pulse oximeter (CMS60D; Contec Medical Systems, Qinhuangdao, China), and a digital voice-activated recorder (Zipcide, Replab Consulting Ltd., Cardiff, UK) (Figure S1). To ensure proper data collection, the primary researcher conducted formal training sessions on device operation. Additionally, volunteers were given a summary information sheet that included the researcher’s contact information for any subsequent inquiries. At the end of the study, participants received a PneumoWave usage questionnaire to assess biosensor comfortability during sleep.

### 2.3. Data Analysis

The procedures for data export and visualization of PneumoWave (PW) have been described previously [18,19]. Prior to analysis, no additional filtering or signal transformation was applied to the combined accelerometer data. Data analysis was categorized into seven main domains: Demographics and Screening Assessment, Data Completeness, Respiratory Rate (RR) Accuracy, Apnea/Hypopnea and Oxygen Desaturation Events, Multimodal Evaluation, Night-to-Night Variation, and Device Evaluation and Usability. All statistical analyses were conducted using SPSS (Version 29.0.1.0, IBM Corp., Armonk, NY, USA). Results are presented as mean ± standard deviation (SD) with 95% confidence intervals (CI), and statistical significance was established at *p* < 0.05.

#### 2.3.1. Demographics Assessments

Participant demographics (age, gender, weight, height, BMI, and ethnicity) and baseline assessments were summarized using standard descriptive statistics.

Snoring from the voice recorder was quantified utilizing the Snoring Episode Index and Snoring Percentage methodologies, as outlined by Kim [22]. Raw audio data from the voice recorder (VR) were processed using MATLAB (Version R2023a, MathWorks, Natick, MA, USA) to isolate snoring sounds from background noise. A bandpass filter (10–100 Hz) was applied to remove low-frequency movement artefacts and high-frequency ambient interference, adhering to American Academy of Sleep Medicine (AASM) recommendations for snoring sound analysis [23]. Although snoring spectral energy can be above 100 Hz, the voice recorder used in the study is a commercial-grade device rather than a validated clinical tool, making it highly susceptible to collecting ambient high-frequency noise. Furthermore, because this device was not clinically validated, voice recordings were manually reviewed to verify true snoring events. Consequently, recorded acoustic data were utilized strictly as a secondary confirmation of snoring, rather than as a formal validation of apnea or hypopnea events. Continuous variables were reported as means with standard deviations, while categorical variables were presented as frequencies and percentages.

#### 2.3.2. Data Completeness

To ensure technical adequacy, rigorous data inclusion criteria were applied in accordance with the AASM guidelines for Home Sleep Apnea Testing (HSAT) [12]. Following these guidelines, only recordings containing a minimum of 4 h of valid data were included in the study, with recordings falling below this threshold systematically excluded from all device analyses. Consequently, valid paired nights criteria are applied, defined as nights where both the investigational device (PneumoWave) and a reference device (i.e., Samsung Galaxy Watch 4 (GW4), Pulse Oximeter (PO), or Voice Recorder (VR)) successfully recorded ≥ 4 h of concurrent data, which were utilized for statistical comparisons. If one device failed to meet this threshold, that specific paired comparison was excluded for that night.

#### 2.3.3. Respiratory Rate Analysis

The biosensor-derived respiratory rate was quantified using the Manual Average Visual Count (MAVC) method, in accordance with previously established studies [18,19]. Upon waveform visualization, individual respiratory cycles were manually identified via direct inspection of the combined signal, utilizing a continuous window that spanned the entire trial duration. To prevent analytical bias, high-amplitude peaks (>0.10 a.u.), identified empirically through visual inspection of the recordings, were excluded from the MAVC calculations as body movement artefacts during sleep. The Samsung Galaxy Watch 4 wearable device, which indirectly uses PPG-derived respiratory (PDR) algorithms based on heart rate variability, was employed as a comparison device of respiratory rate measurements.

Spearman’s correlation coefficient (r) and coefficient of determination (R^2^) was utilized to evaluate the linear relationship between the data derived from the devices. Additionally, a Bland–Altman analysis and an Intraclass Correlation Coefficient (ICC) analysis, a two-way mixed method with absolute agreement, were performed to visualize the limits of agreement.

#### 2.3.4. Apnea, Hypopnea, and Oxygen Desaturation Events

In accordance with AASM criteria [23], apnea events were defined as a >90% drop in peak signal amplitude lasting for at least 10 s. Hypopnea was defined as a ≥3% decrease in saturation of peripheral oxygen (SpO_2_) from baseline lasting a minimum of 10 s, associated with an arousal event.

**Figure 1:**
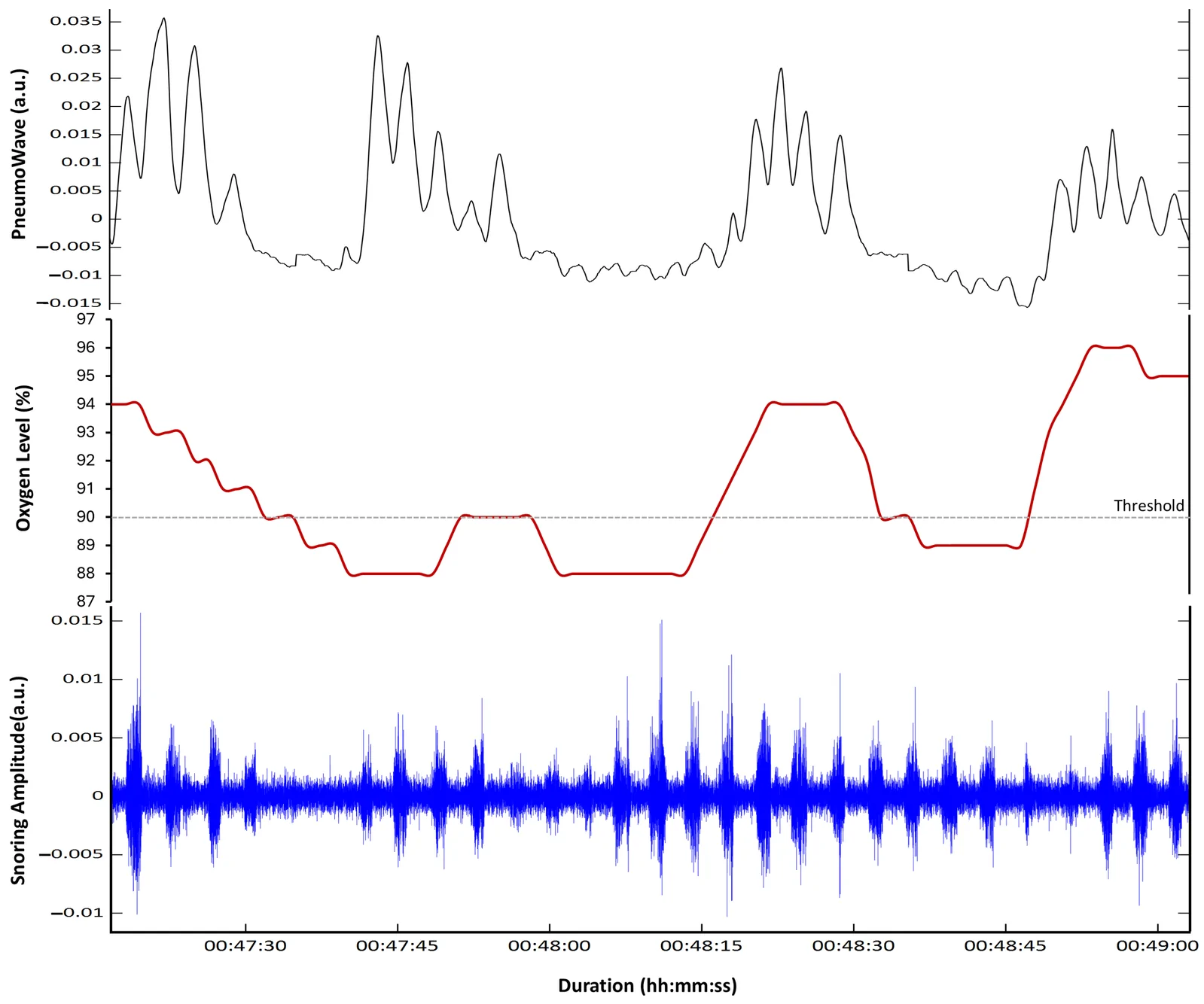
Representative epoch illustrating a severe sleep-disordered breathing event. The synchronized recording demonstrates the physiological cascade of an apneic/hypopneic episode: (**top**) a reduction in respiratory effort captured by the PneumoWave biosensor waveform; (**middle**) the delayed, characteristic transient desaturation in peripheral blood oxygen (SpO_2_), falling below the 90% clinical threshold to a nadir of 88% before recovery; and (**bottom**) the accompanying cessation and subsequent bursting of the acoustic snoring signal.

Data from the portable pulse oximeter (PO) were exported to SpO_2_ Assistant software (Version 3.1.0.4, Contec Medical, Qinhuangdao, China) at the conclusion of the sleep recordings. To synchronize apnea and hypopnea events detected by the biosensor with the oxygen desaturations recorded by the pulse oximeter, a 1-min time window was applied in manual visual scoring from PneumoWave (Figure 1). This 1-min range was deliberately chosen to account for physiological circulation delay (lung-to-finger blood transit time, typically 20–30 s) and instrument response lag associated with peripheral probe placement and signal averaging [24,25]. Based on the Oxygen Desaturation Index (ODI), the severity of OSA was classified as no OSA (ODI < 5 events/h), mild OSA (5 ≤ ODI < 15 events/h), moderate OSA (15 ≤ ODI < 30 events/h), and severe OSA (ODI ≥ 30 events/h), respectively. A similar ODI threshold was applied for AHI.

Data synchronization facilitated the calculation of an Apnea–Hypopnea Index (AHI), the total events of apnea and hypopnea divided by total sleep duration, from the PneumoWave biosensor, which was validated against PO data. The ODI was determined from PO values, which calculate event frequency based solely on valid, artefact-free recording time; this was utilized for analysis. To ensure accuracy, desaturation events were visually verified using strip charts to distinguish physiological hypoxic events from signal artefacts caused by sensor displacement. All definitions of the matrix are summarized in Table 1.

To comprehensively evaluate the device’s performance, statistical analyses were stratified into two primary domains: agreement and diagnostic accuracy. Aggregated measurement agreement and reliability were assessed using Spearman correlation analysis, Intraclass Correlation Coefficients (ICC) (a two-way mixed-effects model with absolute agreement), and Bland–Altman plots. Furthermore, diagnostic performance was quantified via confusion matrices, alongside calculations of sensitivity, specificity, positive predictive value (PPV), negative predictive value (NPV), and the Area Under the Receiver Operating Characteristic Curve (AUC-ROC). ROC curves were generated using continuous PneumoWave AHI values as the test variable and binary ODI thresholds (≥5, ≥15, and ≥30 events/h) as the state variable. Sensitivity, specificity, PPV, and NPV were calculated separately using the predefined clinical AHI thresholds (≥5, ≥15, and ≥30 events/h).

#### 2.3.5. Multimodal Evaluation

The multimodal evaluation was statistically modeled using hierarchical linear regression to assess the incremental diagnostic validity of the PneumoWave biosensor. Control model evaluated the predictive capacity of the subjective baseline (STOP-Bang scores), while Model 1 incorporated the AHI sensor data (PneumoWave metrics) to determine the added variance in predicting OSA severity as defined by the clinical reference (PO), yielding a statistically significant increase in the model’s overall predictive accuracy.

#### 2.3.6. Night-to-Night Variation

To assess night-to-night reliability and longitudinal consistency of physiological metrics across the study period, ICC analysis was employed between data from the devices utilizing a two-way mixed-effects model with a single-measure absolute agreement definition.

#### 2.3.7. Device Evaluation and Usability

At the conclusion of the study, all participants evaluated the PW device using a 5-point Likert scale across five categories: Ease of Use, Comfort, Ease of Attachment and Removal, Clarity of Instructions, and General Convenience. Open-ended questions regarding operational problems and potential improvements were also included. The patient-induced setup error was defined as the inability of the participant to apply the sensor and successfully initiate the recording prior to sleep. Issues occurring during sleep, such as spontaneous device detachment or physical discomfort, were classified separately as usability and wearability events. Quantitative responses were represented on a bar chart, while qualitative feedback from open-ended questions was subjected to thematic analysis.

## 3. Results

### 3.1. Participant Characteristics

A total of 15 participants were included in the study, comprising eight males (53.3%) and seven females (46.7%). The mean age of the cohort was 48.9 ± 14.5 years. The majority of participants identified themselves as White/Caucasian (80%), and the mean BMI was 27.3 ± 4.6 kg/m^2^ (range: 18.5–37.3 kg/m^2^).

Baseline subjective sleep assessments revealed mean STOP-Bang scores of 3.7 ± 1.4. Objective physiological monitoring demonstrated a mean heart rate of 59.2 ± 5.8 beats per minute derived from GW4, and 60.7 ± 6.5 beats per minute from the pulse oximeter (ICC = 0.936, *p* < 0.001). Acoustic analysis yielded a mean snoring percentage of 19.9 ± 21.5% and a snoring index of 7.3 ± 7.0 events/h. A comprehensive summary of the participants’ demographic and clinical characteristics is provided in Table 2.

### 3.2. Data Completeness

Data completeness was evaluated based on the predefined threshold of ≥4 h of valid recording per night. Across all devices used in the study, over 90% of the 45 potential recording nights were successfully captured and available for subsequent analysis. A comprehensive summary of device compliance and data completeness is presented in Table S1.

### 3.3. Respiratory Rate Analysis

**Figure 2:**
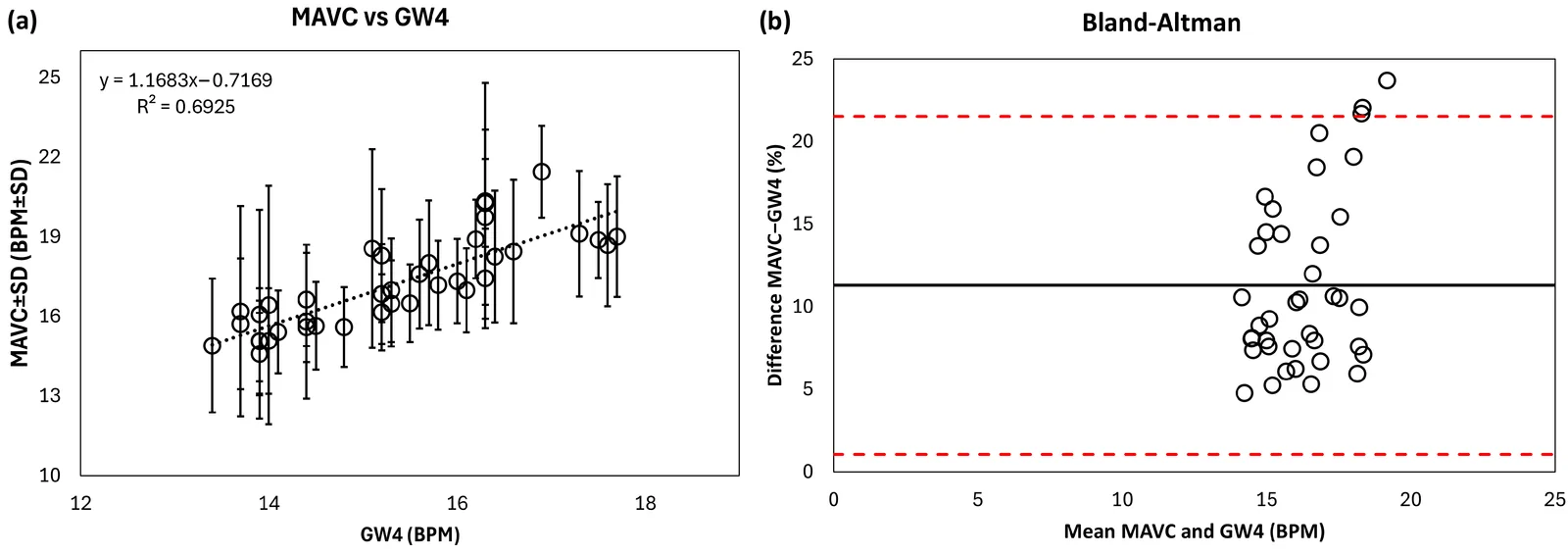
(**a**) Correlation between MAVC and GW4 respiratory rate data and (**b**) the Bland–Altman plot shows the agreement between MAVC and GW4. Solid black lines indicate the mean bias, and dashed red lines represent the 95% limits of agreement (±1.96 SD). MAVC: Manual Average Visual Count, GW4: Galaxy Watch 4.

Following the application of the ≥4-h valid recording threshold, 39 of the 45 available datasets (86.7%) were included in respiratory rate analysis. The Manual Average Visual Count derived from the PW biosensor was compared against the GW4 reference data. The mean respiratory rate was 17.2 ± 1.7 breaths per minute (BPM) for the PW device and 15.3 ± 1.2 BPM for GW4. Correlation analysis demonstrated a strong positive relationship between the two devices (*r* = 0.87, R^2^ = 0.69, *p* < 0.001; Figure 2a). Furthermore, ICC analysis indicated moderate agreement 0.612, *p* < 0.001 (Table S2). Finally, Bland–Altman analysis (Figure 2b) revealed a mean bias of 1.87 between measurements, with the 95% limits of agreement ranging from a lower limit of −0.95 to an upper limit of 2.89.

### 3.4. Apnea, Hypopnea, and Oxygen Desaturation Events

Analysis of valid PO data revealed a mean basal SpO_2_ of 95.8 ± 1.5% and a nadir SpO_2_ of 85.5 ± 5.2%. Over the 3-night study period, the average total number of SpO_2_ desaturation events was 302.2 ± 279.6, corresponding to a mean ODI of 13.4 ± 11.4 events/hour. Time < 90% of PO reference was 10.1 ± 17.1 min with high inter-participant variability. A comprehensive summary of the oxygen saturation profiles and related events is provided in Table 3.

**Figure 3:**
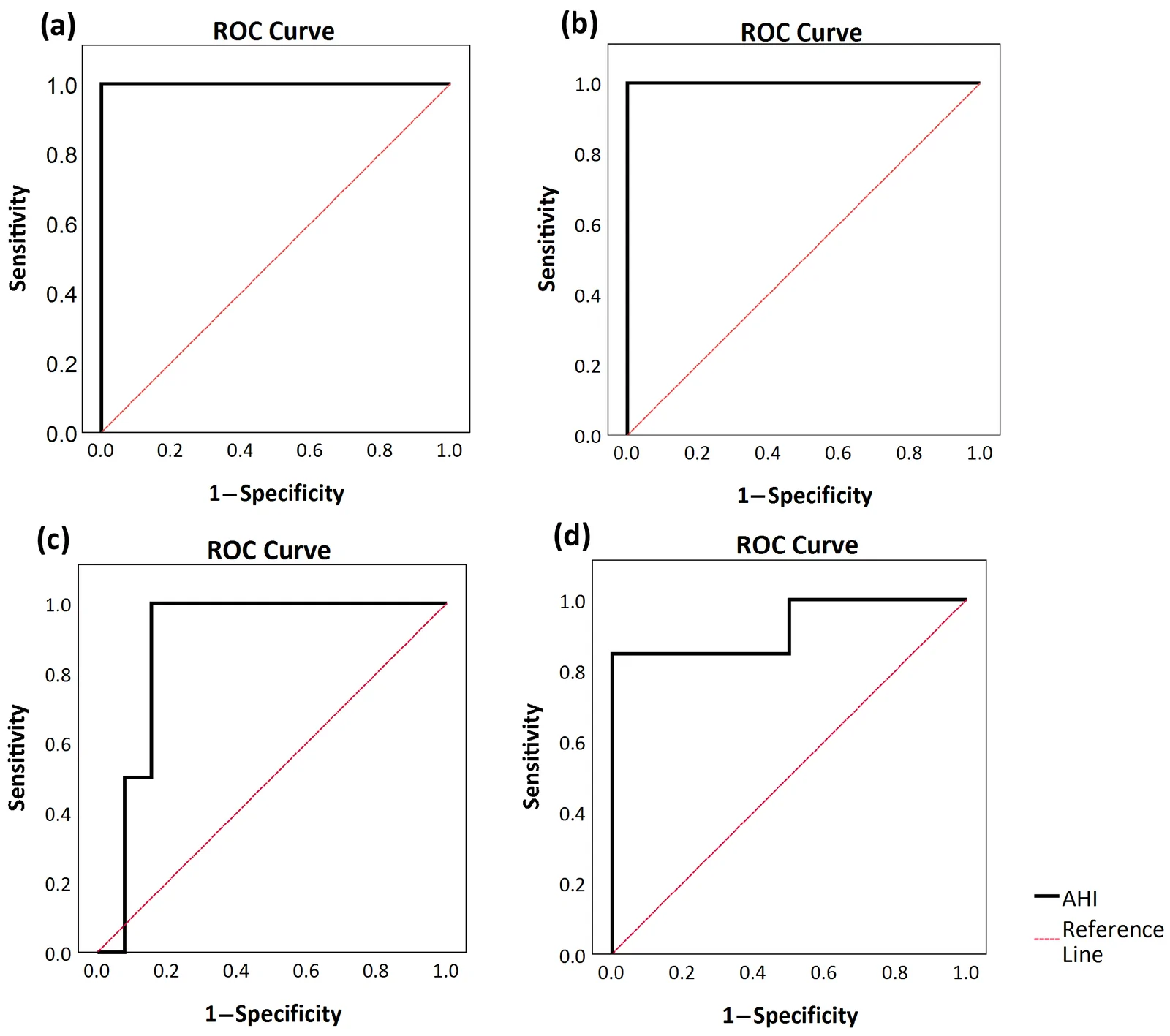
(**a**) ROC curve AHI (black) from PneumoWave with ODI ≥ 5 events/hour; (**b**) ROC curve AHI (black) from PneumoWave with ODI ≥ 15 events/hour; (**c**) ROC curve AHI (black) from PneumoWave with ODI ≥ 30 events/hour; (**d**) ROC curve AHI (black) from PneumoWave with STOP-Bang ≥ 3. ROC: Receiver Operating Characteristic Curve.

Table 4 summarizes sensitivity and specificity metrics according to cutoff points from ODI and the STOP-Bang questionnaire. As seen in Figure 3, diagnostic accuracy of AHI from PneumoWave was high for all severity levels (AUC ≥ 0.89).

### 3.5. Multimodal Analysis

In the multimodal analysis, a hierarchical linear regression model was employed to determine the incremental predictive capacity of the PneumoWave biosensor metrics for estimating ODI. The baseline model, incorporating the STOP-Bang questionnaire, yielded an R^2^ of 0.177 (*p* = 0.118). In Model 1, the AHI derived from the PneumoWave biosensor was introduced, resulting in an R^2^ of 0.907 (*p* < 0.001; Table 5).

### 3.6. Night-to-Night Variation

To evaluate night-to-night reliability, ICC analysis was performed across the three recording nights for all diagnostic parameters, including ODI, Snoring Index, Snoring Percentage, and AHI. The analysis demonstrated excellent inter-night reliability for all metrics, with ICC values exceeding 0.93 (*p* < 0.001) (Table S3).

### 3.7. PneumoWave Biosensor Usability

**Figure 4:**
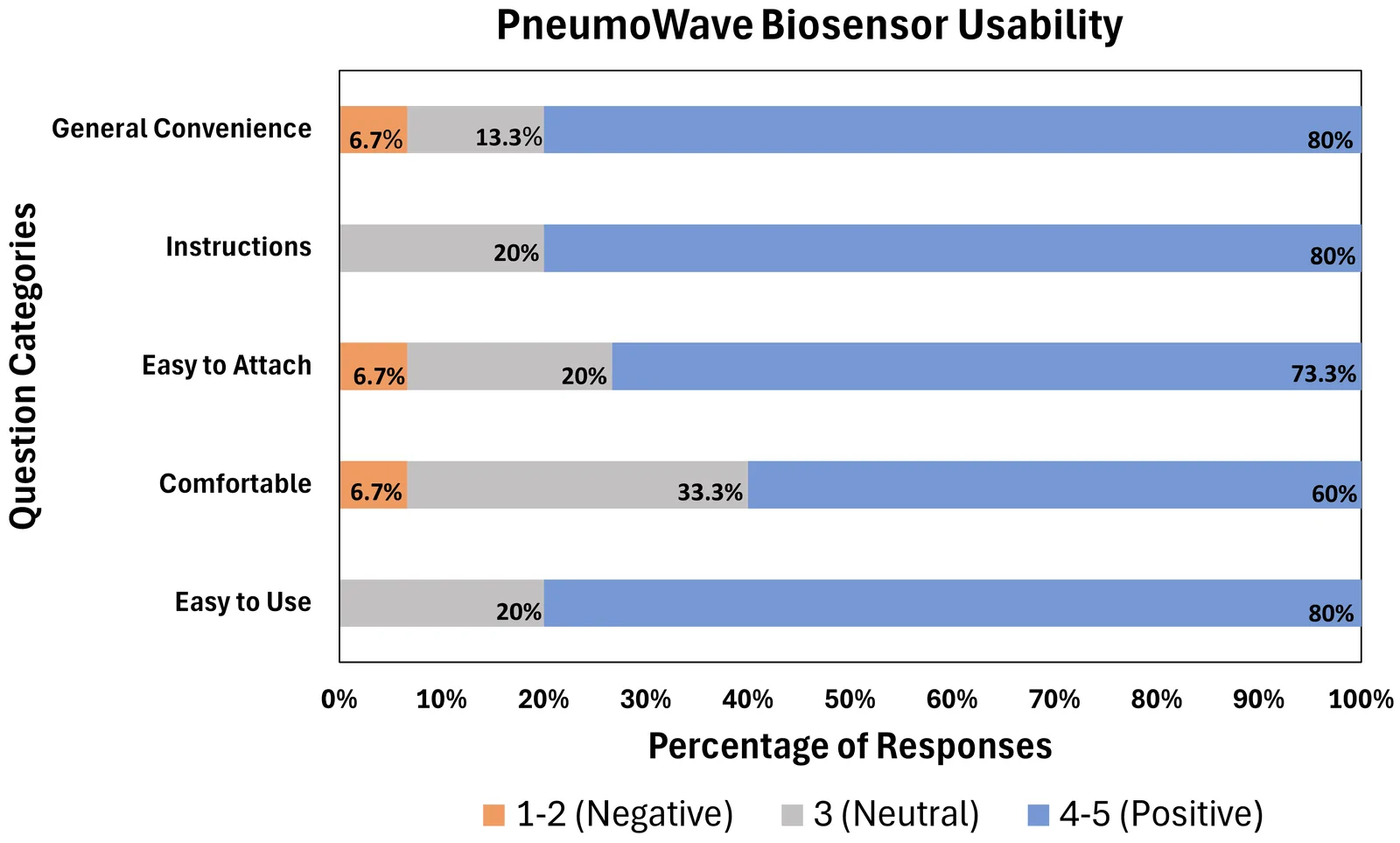
Subjective usability assessment of the PneumoWave biosensor. Participants (*n* = 15) evaluated the device using a 5-point Likert scale (1 = Strongly Disagree, 5 = Strongly Agree). Data are presented as the percentage distribution of responses, diverging from a neutral midpoint (3). Orange segments indicate negative responses (1–2), gray segments represent neutral responses (3), and blue segments indicate positive responses (4–5).

Following the end of the study, participants completed the PneumoWave Device Evaluation form to assess the usability and comfort of the biosensor. As illustrated in Figure 4, the device received predominantly positive feedback, with over 60% of participants expressing satisfaction across all evaluation domains.

During initial device usage, there were no patient-induced setup errors; all participants successfully positioned the sensor and initiated the recordings. In the qualitative assessment, 12 out of 15 participants reported encountering minor technical or comfort-related challenges. The most frequently cited issues included difficulty falling asleep due to the device’s physical presence or indicator lights, tablet interface limitations, such as authentication errors and data transmission issues, and skin irritation or discomfort during adhesive tape removal. Additionally, a small subset of participants noted the device detached during sleep or had difficulties with ECG electrode placement. Individual reports also highlighted ergonomic challenges for stomach sleepers, the inconvenience of manual battery insertion, and a perceived complexity in the device setup process. However, 11 of 15 participants explicitly praised the biosensor for its lightweight profile, compact size, and overall ease of operation.

## 4. Discussion

### 4.1. Respiratory Rate Measurement

Respiratory rate (RR) measurements between the PneumoWave biosensor and the Galaxy Watch 4 demonstrated a strong correlation. Furthermore, intraclass correlation coefficient analyses revealed good agreement between the devices, despite their differing measurement methodologies. These findings are consistent with previous validation studies, in which the PneumoWave biosensor exhibited good agreement against both mechanical ventilators and direct observational counting, with a bias of −0.04 BPM in the supine position [18,19]. Similarly, the Galaxy Watch 4/5 has been validated against PSG for overnight RR monitoring, reporting a root mean square error of 1.13 BPM. However, literature suggests that the accuracy of Galaxy Watch RR measurements decreases in the presence of severe AHI [26]. In the present study, the mean difference in RR between the PneumoWave biosensor and the Galaxy Watch 4 was 1.9 BPM which falls well within clinically acceptable limits for continuous overnight monitoring.

Although the PneumoWave biosensor and the Galaxy Watch 4 utilize fundamentally different sensing modalities, direct measurement of thoracic wall motion versus indirect respiratory estimation from heart rate variability (HRV), respectively, our findings show they reach a strong consensus on overnight respiratory rate. The technical robustness of the PneumoWave biosensor was evidenced by its consistent performance across diverse benchmarks, ranging from controlled mechanical ventilation [15] and direct observational counts [16] to wrist-based heart rate algorithms. That this level of reliability was maintained even in the uncontrolled environment of a home sleep assessment further highlights the strength and maturity of the biosensor’s signal processing to perform effectively outside traditional clinical settings.

### 4.2. Apnea, Hypopnea, and Oxygen Desaturation Events

This study evaluated the diagnostic and screening performance of the PneumoWave biosensor against pulse oximetry and the STOP-Bang questionnaire. Regarding screening accuracy, STOP-Bang and PneumoWave metrics did not show any significant correlation. However, ROC analysis showed that AHI metrics and STOP-Bang scores show accuracy (AUC = 0.92).

Concerning diagnostic accuracy, the PneumoWave metrics and ODI from pulse oximetry showed a good correlation and agreement (*r* > 0.85, *p* < 0.001). The AHI-based metric demonstrated high diagnostic utility, achieving an AUC of 1.00 for detecting both mild (≥5 events/h) and moderate (≥15 events/h) OSA, with a slight decline to 0.89 at the severe threshold (≥30 events/h). Although the sensitivity of the biosensor decreased when the severity of OSA increased, the specificity remained constant which strengthens the diagnostic ‘rule-in’ capacity of it as a clinical tool. When coupled with a robust PPV, a positive result reliably confirms the presence of the disease, thereby minimizing the risk of false-positive clinical referrals. This high specificity ensures that healthcare resources are directed efficiently toward true-positive cases.

Furthermore, a limitation of type IV single-channel devices is their restricted ability to accurately diagnose central sleep apnea, a condition characterized by the cessation of breathing without accompanying respiratory effort [27]. Unlike conventional single-channel systems, the PneumoWave biosensor has an integrated triaxial accelerometer to directly measure chest wall mechanics. This provides a distinct advantage in capturing the absence of respiratory effort characteristic of central sleep apnea, a capability we have previously demonstrated in vitro [18].

### 4.3. Multimodal Analysis

A pivotal aspect of our study was the diagnostic synergy achieved by combining the STOP-Bang questionnaire with continuous chest wall movement data derived from the PneumoWave biosensor. While the STOP-Bang questionnaire is widely utilized as a first-line screening tool due to its high diagnostic sensitivity, it is inherently limited by low specificity, which frequently leads to false-positive referrals [10,12]. Our findings suggest that this streamlined two-step approach, combining a validated clinical risk screening tool with continuous chest wall motion monitoring, has the potential to improve the diagnostic assessment of OSA in home settings and enables a shift from a ‘one-size-fits-all’ approach towards stratified medicine. By categorizing patients according to disease severity or their likelihood of adverse outcomes, healthcare interventions can be targeted more effectively. Ultimately, this strategy has the potential to substantially alleviate the clinical burden and prolonged waiting lists associated with in-laboratory polysomnography, ensuring that those most likely to benefit receive timely and appropriate treatment. However, high R^2^ observed in multimodal analysis could be the result of physiological coupling between breathing effort and oxygen desaturation events. The high correlation between AHI and ODI may highlight this physiological cascade.

### 4.4. Night-to-Night Variation and PneumoWave Biosensor Usability

In traditional OSA diagnostics, reliance on single-night polysomnography is frequently associated with misclassification rates ranging from 20% to 50% due to significant night-to-night variability [28]. ICC revealed consistent agreement across all physiological parameters over the three-night monitoring period using the PneumoWave biosensor and this offers the potential to reduce the likelihood of patient miscategorization.

In terms of usability, most participants reported that the PneumoWave biosensor was comfortable to wear and easy to apply and remove, with only minor usability issues reported. A well-documented limitation of traditional home sleep testing is the high incidence of patient-related setup errors in unsupervised environments [27]. While four nights of data from PneumoWave were lost during the present study, none of these instances were attributable to patient error, but were entirely due to technical device faults. In contrast, data loss associated with comparator devices was heavily reliant on patient behavior and physical constraints; for instance, pulse oximetry data loss was caused by sensor detachment from the finger during sleep, while data loss from the smartwatch and voice recorder resulted from battery depletion.

The robust longitudinal consistency observed with the PneumoWave biosensor not only mitigates the risk of misclassification but also strongly indicates high patient tolerability. Although a small proportion of participants, particularly stomach sleepers, reported mild discomfort, objective measurements demonstrated stable performance with no significant night-to-night variation. This stability indicates that despite minor subjective concerns, it did not induce a measurable first-night effect, ultimately allowing for the consistent capture of sleep architecture.

Although some degree of data loss is inevitable in both conventional home sleep apnea devices and wearable technologies, no user-induced failures were observed with the PneumoWave biosensor, reflecting its intuitive design and straightforward application. These findings support the suitability of the device for self-administered home sleep monitoring. This highlights a significant operational advantage, demonstrating that the device is highly suitable for self-administered home monitoring.

### 4.5. Strengths

A primary strength of this study is its longitudinal, real-world design. By monitoring participants over three consecutive nights in an uncontrolled home environment, the study effectively mitigated the first-night effect that accounts for the night-to-night variability that frequently conflicts with single-night diagnostic assessments. Furthermore, the robust usability of the biosensor, demonstrated by the absence of patient-induced setup errors, highlights its operational viability for unsupervised screening. Clinically, the study’s multimodal approach was a significant asset. By pairing a validated clinical tool (the STOP-Bang questionnaire and pulse oximetry) with continuous chest wall monitoring, this research moves beyond standard oximetry.

By demonstrating alignment between initial screening questionnaires and diagnostic device, the PneumoWave biosensor could bridge a critical gap in the sleep medicine pathway. The lightweight profile and ease of application of the PneumoWave biosensor, coupled with its capacity to accurately stratify patients, make it an optimal tool for primary diagnostic triage. Furthermore, its consistent night-to-night stability positions it as an effective longitudinal monitoring solution for patients undergoing OSA therapy.

### 4.6. Limitations

This study had several potential limitations. First, validation was conducted in an uncontrolled home environment utilizing a single-channel sleep apnea test without concurrent in-laboratory polysomnography. While this design authentically reflects real-world clinical application, the absence of a gold-standard diagnostic tool means our findings rely on comparisons with surrogate markers from pulse oximetry and wearable devices, rather than a direct, epoch-by-epoch comparison against comprehensive EEG and standard airflow sensors.

Secondly, most participants had self-reported breathing problems, which introduces potential selection bias. Since our cohort consisted of individuals at elevated risk for OSA (STOP-Bang ≥ 3), the exceptionally strong diagnostic agreement observed in this study may be partially driven by the enrichment process adopted and might not generalize to lower-risk populations. Additionally, because the cohort was not stratified by demographic or physiological factors like age and BMI, we are limited in our ability to evaluate how specific patient traits might alter respiratory signal pattern and overall device accuracy. Furthermore, the evaluation of respiratory rate and apnea/hypopnea events relied exclusively on researcher interpretation which may introduce a degree of observer-dependent bias.

Although our findings demonstrated an absence of patient-induced setup errors, a minor degree of data loss (8.9%) still occurred due to technical hardware faults. This highlights the ongoing need for continued hardware and software optimization for future studies to ensure absolute data continuity in decentralized sleep monitoring.

Furthermore, as Type IV screening devices lack an electroencephalography (EEG) sensor, we cannot definitively differentiate true sleep from quiet wakefulness. Consequently, the calculation of event indices relies on total valid recording time rather than true total sleep time. This reliance on recording time may inherently inflate or deflate the calculated event rates depending on the unmeasured duration of wakefulness during the night.

Finally, a physiological limitation can arise from the nature of OSA, which can be characterized by continuous respiratory effort during a collapse airway. Since the biosensor detects chest movements using a triaxial accelerometer, chest movements during OSA episode may reduce the capture of true apnea or hypopnea events by the biosensor. For true event detection, concurrent measurement during OSA with airflow sensors is required.

## 5. Conclusions and Future Directions

This study evaluated the feasibility of the PneumoWave, a triaxial accelerometer, in people who self-reported breathing problems in the home environment, comparing it to a wearable and a single-channel sleep device. These findings suggest that the PneumoWave biosensor may serve as a practical and low-cost home-based screening tool to support patient triage and prioritization for polysomnography, offering a simple self-administered alternative to conventional multi-sensor systems.

To build upon the current findings, future research will prioritize comprehensive clinical validation, incorporating concurrent in-laboratory PSG alongside larger-scale and multi-night HSAT deployments in the home environment. Subsequent trials will aim to include larger, well-defined clinical cohorts consisting of patients formally diagnosed with varying severities of OSA.

Furthermore, given the high sensitivity of triaxial accelerometers to physical motion, the biosensor holds potential to serve as a valuable proxy monitor for sleep arousals. Future studies should conduct comparative analyses with concurrent EEG to validate these movement-based physiological events against true cortical arousals, thereby further exploring their utility in assessing increased respiratory effort, gasping, and restless sleep.

In future work, machine learning systems such as lightweight attention domain-adaptive temporal convolutional network models [29] and backpropagation neural network architecture [30] can be employed to reduce manual operator assessment, accelerating assessment workflow. However, a critical methodological objective for future iterations is the development of a transparent, glass-box diagnostic algorithm. Rather than relying on a fully automated black-box system, this approach will facilitate manual over-reading and validation of respiratory events by healthcare professionals, ensuring clinical oversight and fostering diagnostic trust.

## Figures and Tables

**Table 1:** Definition of metrics. AHI: Apnea–Hypopnea Index; ODI: Oxygen Desaturation Index.

Terms	Definition	Reference
Apnea	A minimum 90% reduction in peak signal excursion from the pre-event baseline, sustained for at least 10 s	[23]
Hypopnea	A reduction in peak signal excursion of at least 30% from baseline, sustained for at least 10 s, and associated with either a ≥3% oxygen desaturation or an arousal.	
AHI	Summing the total number of apnea and hypopnea events and dividing by the total sleep time from PneumoWave biosensor	
ODI	Quantifying the total number of desaturation events per hour of sleep from Pulse oximetry	
Snoring Episode Index	Dividing the total number of identified snoring episodes by the total sleep time	[22]
Snoring Percentage	Calculating the ratio of the cumulative duration of all snoring events to the total sleep time	

**Table 2:** Demographic characteristics of all sleep study participants. PO: Pulse Oximetry; GW4: Galaxy Watch 4; SD: Standard Deviation.

Variables	Characteristic	Value *
**Age (years)**	Mean ± SD	48.9 ± 14.5 (29–76)
**Weight (kg)**		81.4 ± 15.4 (54–115)
**Height (cm)**		172.9 ± 12.2 (157–193)
**BMI (kg/m^2^)**		27.3 ± 4.6 (18.5–37.3)
**Gender**	Male Female	8 (53.3%) 7 (46.7%)
**Ethnicity**	White/Caucasian Asian/Asian British Other Prefer not to say	12 (80%) 1 (6.67%) 1 (6.67%) 1 (6.67%)
**STOP-Bang**	Average score Low (<3) Intermediate risk (3–4) High risk (>4)	3.7 ± 1.4 2 (13.4%) 8 (53.3%) 5 (33.3%)
**Heart Rate** **PO** **GW4**	Mean ± SD	60.7 ± 6.5 59.2 ± 5.8
**Snoring Percentage**	Mean ± SD	19.9 ± 21.5
**Snoring Index**	Mean ± SD	7.3 ± 7.0

* Data represented as mean ± standard deviation (minimum–maximum) for continuous variable and as *n* (%) for categorical variables.

**Table 3:** Summary of Oxygen Saturation Level and Events from the Pulse oximetry device. SpO_2_: Oxygen saturation; SD: Standard deviation; ODI: Oxygen Desaturation Index; AHI: Apnea–Hypopnea Index; CI: Confidence Interval; ICC: Intraclass Correlation; LOA: Limits of Agreement; *p*: *p*-value.

	Mean ± SD		Minimum–Maximum
I. Baseline information			
**Basal SpO_2_**	95.8 ± 1.5		93.3–97.7
**Nadir SpO_2_**	85.5 ± 5.2		77–95
**Total Events SpO_2_ (count)** **99–95% Events** **94–90% Events** **89–85% Events** **84–80% Events** **79–75% Events** **74–70% Events** **69–65% Events** **64–60% Events**	302.2 ± 279.6		0–907
	68.1 ± 56.6		0–206
	208.3 ± 223.1		0–723
	24.1 ± 49.4		0–183
	1.1 ± 2.2		0–8
	0.5 ± 1.6		0–6
	0.3 ± 1.3		0–5
	0		0
	0.1 ± 0.5		0–2
**ODI (events/hour)** **ODI < 5** **5 ≥ ODI > 15** **15 ≥ ODI > 30** **ODI ≥ 30**	13.4 ± 11.4		0.7–34.4
	4 (26.7%)		0.4–4.3
	6 (40%)		5.4–14.1
	3 (20%)		18.7–29.3
	2 (13.3%)		30.9–35.1
**Time < 90% (Minute)**	10.1 ± 17.1		0–64
**AHI (events/hour)** **AHI < 5** **5 ≥ AHI > 15** **15 ≥ AHI > 30** **AHI ≥ 30**	7.2 ± 6.3		0.7–21.1
	8 (53.3%)		0.7–4.7
	5 (33.3%)		6.0–14.5
	2 (13.3%)		15.9–21.1
	0		0
**II. Screening Accuracy**			
	**Correlation (95% CI, *p*)**		
**AHI vs. STOP-Bang**	0.294 (–0.273–0.709, *p* = 0.288)		
**III. Diagnostic Accuracy**			
	**Correlation** **(95% CI, *p*)**	**ICC** **(95% CI, *p*)**	**Agreement** **(95% LOA)**
**AHI vs. ODI**	0.964 (0.890–0.989, *p* < 0.001)	0.793 (0.015–0.942, *p* < 0.001)	6.15 −5.43–17.73

Due to non-normal distribution of the AHI dataset as confirmed by the Shapiro–Wilk test (*p* < 0.05), Spearman’s rank correlation coefficient was utilized to assess the association between diagnostic indices, ensuring the robustness of the analysis against potential outliers. Correlation between ODI and AHI was 0.964 (*p* < 0.001). The ICC analysis revealed agreement between ODI vs. AHI (0.793, *p* < 0.001). Bland–Altman analysis showed that mean bias was 6.15 for ODI vs. AHI (Table 3-Section II).

**Table 4:** Sensitivity, specificity, PPV, and NPV analysis at 5, 15, and 30 cutoff levels (ODI) and 3 cutoff (STOP-Bang) for AHI from PneumoWave. AHI: Apnea–Hypopnea Index deprived from PneumoWave; AUC: Area under the Curve; PPV: Positive Predictive Value; NPV: Negative Predictive Value; N/A: Not Applicable.

	Diagnostic Metrics	ODI ≥ 5 (Mild)	ODI ≥ 15 (Moderate)	ODI ≥ 30 (Severe)	STOP-Bang ≥ 3 * (Intermediate–High Risk)
**AHI**	**AUC**	1.00	1.00	0.89	0.92
	**Sensitivity**	0.64	0.40	0	0.54
	**Specificity**	1.00	1.00	1.00	1.00
	**PPV**	1.00	1.00	N/A **	1.00
	**NPV**	0.50	0.77	0.87	0.25

* AHI ≥ 5 was accepted as cutoff value. ** Positive Predictive Value (PPV) could not be calculated because no true or false positive events were predicted by the device at this threshold, resulting in a denominator of zero.

**Table 5:** Hierarchical regression analysis demonstrated the incremental validity of the PneumoWave biosensor in predicting OSA severity (ODI).

Model/Block	Variables	R^2^	ΔR^2^	Sig. F Change
**Baseline Model**	STOP-Bang	0.177	0.177	0.118
**Model 1**	STOP-Bang + AHI	0.907	0.730	<0.001

## Data Availability

Data are available upon request by contacting the corresponding author.

## Supplementary Materials

The following supporting information can be downloaded at https://www.mdpi.com/article/10.3390/bios16080454/s1, Figure S1: Recording schema with PneumoWave biosensor, wearable watch and portable pulse oximetry and voice recorder during sleep; Table S1: Data completeness from devices; Table S2: Average respiratory rate from the PneumoWave device and the Samsung Galaxy Watch 4. SD: Standard deviation; *p*: *p*-value; Δ: Mean Difference; *r*: Correlation coefficient; ICC: Intraclass Correlation; LOA: Limits of Agreement; Table S3: Intraclass Correlation during 3 nights for all diagnostic parameters.

## Abbreviations

The following abbreviations are used in this manuscript:

Δ	Mean Difference
AASM	American Academy of Sleep Medicine
AHI	Apnea–Hypopnea Index
AUC	Area Under the Curve
AUC-ROC	Area Under the Receiver Operating Characteristic Curve
BMI	Body Mass Index
BPM	Breaths per minute
CI	Confidence Interval
EEG	Electroencephalography
GDPR	General Data Protection Regulation
GW4	Galaxy Watch 4
HRV	Heart rate variability
HSAT	Home Sleep Apnea Testing
ICC	Intraclass Correlation Coefficients
LOA	Limits of Agreement
MAVC	Manual Average Visual Count
NPV	Negative predictive value
ODI	Oxygen Desaturation Index
OSA	Obstructive sleep apnea
*p*	*p*-value
PO	Pulse Oximetry
PPV	Positive predictive value
PSG	Polysomnography
PW	PneumoWave
*r*	Correlation coefficient
RR	Respiratory rate
SD	Standard Deviation
SpO_2_	Saturation of peripheral oxygen
UEC	University Ethics Committee
VR	Voice Recorder
