# Supplementary material for: A Wearable Respiratory Monitor for Home-Based Screening and Stratification of Obstructive Sleep Apnea: A Pilot Study in Participants with Intermediate-to-High STOP-Bang Scores
Source: Biosensors (Basel). 2026 Aug 20;16(8):454. doi: 10.3390/bios16080454 (PMC13511218; doi:10.3390/bios16080454)
Supplement: Supplementary file 1 [file biosensors-16-00454-s001.zip › biosensors-4461360-supplementary.pdf]

biosensors

MDPI

Supplementary Materials

A Wearable Respiratory Monitor for Home-Based Screening and Stratification of Obstructive Sleep Apnea: A Pilot Study in Participants with Intermediate-to-High STOP-Bang Scores

Burcu Kolukisa Birgec, Beyza Toprak and Alexander Balfour Mullen*

Strathclyde Institute of Pharmacy and Biomedical Sciences, University of Strathclyde, Glasgow G4 0RE, UK; burcu.kolukisa@strath.ac.uk (B.K.B.); beyza.toprak.2022@uni.strath.ac.uk (B.T.)
* Correspondence: a.mullen@strath.ac.uk

List of Materials

Recruitment Information……………………………………………………………………1
Figure S1. Recording schema with PneumoWave biosensor, wearable watch and portable pulse oximetry and voice recorder during sleep……………………………………2
Table S1. Data completeness from devices………………………………………………3
Table S2. Average respiratory rate from the PneumoWave device and the Samsung Galaxy Watch 4...……………………………………………………………………………4
Table S3. Intraclass Correlation during 3 nights for all diagnostic parameters………………………...5

Recruitment Information

Inclusion Criteria:

- Patients aged over 18 years and males and females living in the Glasgow area and available for face-to-face meetings
- Have a snoring problem or complain of breathing problems during sleep that they/their partner or family or friend notice
- Who has prior experience with the diagnostic pathway for sleep apnoea or related sleep-breathing disorders
- Are willing and able to give informed assent/consent and available for study visits due to device training and technical problems
- Be able to use a ‘smart devices’
- Able to comply with all study requirements
- Can speak and read English

Exclusion Criteria:

- Unable to provide consent
- Patients with recent or current CPAP or other sleep masks therapy use now or in the past 3 months
- Patients with sleep medication in the past 3 months such as zopiclone, zolpidem, melatonin, benzodiazepines, amitriptyline, antihistamines or valerian extract

- Patients who have any cardiovascular, pulmonary diseases, systemic or other organ-related diseases
- Patients with pregnancy
- Patients already involved in an ongoing research study
- Patients with a known allergy to surgical adhesive tape or straps
- Patients who have an unstable sleep environment that may be disrupted by external factors such as a co-sleeping partner, children or pets that could interfere with sleep

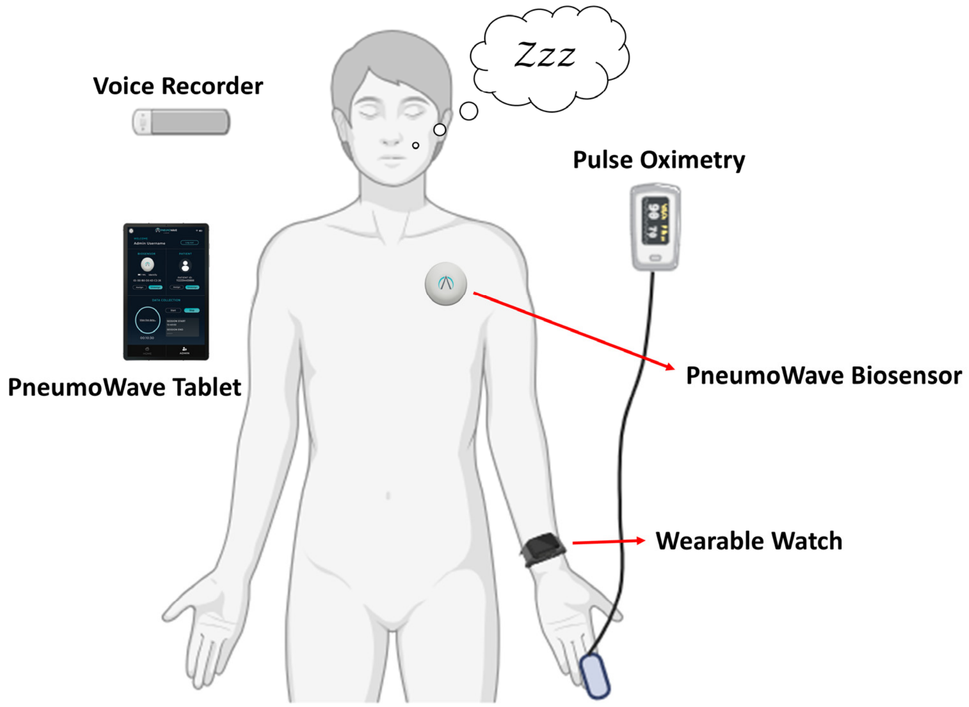

Figure S1. Recording schema with PneumoWave biosensor, wearable watch and portable pulse oximetry and voice recorder during sleep.

Table S1. Data completeness from devices.

	**Total Nights (N)**	**Successful Nights (≥ 4 h)**	**Failed Nights (< 4 h)**	**Compliance Rate (%)**	**Failure Reason**
Pulse Oximetry	45	43	2	95.6%	Probe displacement
PneumoWave		41	4	91.1%	Signal Loss
Galaxy Watch 4		44	1	97.8%	Battery depletion
Voice Recorder		44	1	97.8%	Battery depletion

Table S2. Average respiratory rate from the PneumoWave device and the Samsung Galaxy Watch 4.SD: Standard deviation; p: p-value; Δ: Mean Difference; r: Correlation coefficient; ICC: Intraclass Correlation; LOA: Limits of Agreement.

	**Mean (± SD)**	**Δ (± SD)**	**r (%95 CI, p)**	**ICC (95% CI, p)**	**Agreement (95% LOA)**
**PW**	17.2 ± 1.7	1.9 ± 1.0	0.870	0.612	1.87

**GW4**	15.3 ± 1.2	(0.759 - 0.931, p < 0.001)	(–0.198 - 0.875, p < 0.001)	(–0.95 - 2.89)

Table S3. Intraclass Correlation during 3 nights for all diagnostic parameters.

**Variable**	**ICC**	**95% CI**	**P-value**
ODI	0.981	0.953 - 0.994	<0.001
Snoring Percentage	0.934	0.839 - 0.977	<0.001
Snoring Index	0.934	0.849 - 0.975	<0.001
AHI	0.956	0.868 - 0.989	<0.001

https://doi.org/10.3390/xxxxx
